# Iron-mediated remediation of arsenic-induced suppression of root morphogenesis and radial oxygen loss in wetland plant

**DOI:** 10.3389/fpls.2025.1736435

**Published:** 2025-12-19

**Authors:** Sitong Jiang, Zhengyu Zhu, Yitong Pan, Rongrong Shi, Zhixi Wang, Mohammad Mazbah Uddin, Song Wang, Jingchun Liu, Kang Mei

**Affiliations:** 1 Jiangsu Institute of Marine Resources Development, Jiangsu Key Laboratory of Marine Bioresources and Environment, Jiangsu Ocean University, Lianyungang, China; 2 State Key Laboratory of Marine Environmental Science, College of Ocean and Earth Sciences, College of the Environment and Ecology, Xiamen University, Xiamen, China; 3 Department of Civil and Environmental Engineering, Princeton University, Princeton, NJ, United States; 4 Key Laboratory of the Ministry of Education for Earth Surface Processes & College of Urban and Environmental Sciences, Peking University, Beijing, China; 5 Second Institute of Oceanography, Ministry of Natural Resources (MNR), Hangzhou, China

**Keywords:** arsenic-iron interaction, radial oxygen loss, rice seedlings, root morphogenesis, wetland plants

## Abstract

Arsenic (As) contamination in paddy soils disrupts root morphogenesis and radial oxygen loss (ROL), yet the dynamic interplay between iron (Fe) plaque formation and plant physiological adaptation remains poorly understood across temporal scales. This study investigated Fe-mediated mitigation of inorganic As toxicity through hydroponic experiments with varying As (0, 10, and 30 μmol/L) and Fe (0–100 μmol/L) concentrations over exposure periods ranging from acute to chronic scenarios. Pot experiments revealed that 1-week high-As (HAs) exposure reduced chlorophyll content by 28% and suppressed ROL by 50% compared with the control group (CK), whereas prolonged low-As (LAs) exposure induced partial chlorophyll recovery (LAs > CK > HAs). Fe supplementation enhanced root biomass by 1.18–2.39 times under chronic As stress and significantly increased ROL rates by 1.38–2.83 times through Fe-plaque formation, which sequestered 38%–52% of root-associated As. Root porosity peaked at 9.84% under As–Fe co-treatment but showed an inverse correlation with ROL. Anatomical analysis revealed Fe-driven restoration of aerenchyma (46.32% cross-sectional area) and reinforcement of lignification barriers. These findings highlight Fe’s dual role in alleviating As toxicity through physical immobilization and physiological adaptation, offering insights into Fe-based remediation strategies for As-contaminated wetlands.

## 1 Introduction

Arsenic (As) contamination in wetland ecosystems, particularly rice paddies, represents a pervasive threat to global food security and public health. With more than 100 million people worldwide exposed to As through contaminated rice consumption, the urgency to understand and mitigate its impacts has never been greater (Dong et al., 2022; Guo et al., 2020; Johnston et al., 2011; Zhao et al., 2010) In flooded paddy soils, As undergoes complex biogeochemical transformations in which iron–sulfur cycling, organic matter dynamics, and microbial activity collectively govern its speciation and mobility (Mukherjee et al., 2024; Shi et al., 2020). The resulting inorganic species (As³^+^ and As^5+^) exhibit high bioavailability, disrupting essential physiological processes in wetland plants such as rice (*Oryza sativa* L.), including root morphogenesis, radial oxygen loss (ROL), and nutrient acquisition (Hasanuzzaman et al., 2015; Suda and Makino, 2016). This disruption not only compromises plant growth and productivity but also facilitates As entry into the food chain, creating a critical environmental health challenge.

The root–soil interface serves as the primary site of As dynamics, where plant roots actively modify their immediate environment through the exudation of low-molecular-weight organic acids and dissolved organic carbon (Jiang et al., 2023; Mei et al., 2023, Mei et al., 2022; Uddin et al., 2024) These exudates alter sediment pH, redox potential, and mineral surface properties, simultaneously influencing As mobilization and immobilization (Jia et al., 2013; Liu et al., 2025; Mei et al., 2020; Zheng et al., 2013). Particularly significant is the iron plaque—a complex mixture of iron (oxyhydr)oxides precipitated on root surfaces—which can sequester substantial amounts of As through adsorption and co-precipitation (Mei et al., 2021; Zhang et al., 2017). However, the efficacy of this natural barrier varies considerably with environmental conditions, root physiology, and As exposure regimes. Although numerous studies have characterized these processes in soil systems, the intricate interplay of multiple factors often obscures causal relationships, highlighting the need for controlled investigations to isolate key mechanisms.

Hydroponic systems offer a strategic approach to dissect fundamental plant physiological responses to As stress and Fe-mediated mitigation, free from the confounding variables inherent in soil environments. Although hydroponic conditions may not fully replicate field scenarios—particularly regarding microbial diversity, soil texture, and organic matter interactions—they provide unparalleled precision in controlling ion concentrations and monitoring temporal changes in root physiology (Cao et al., 2024; Liu et al., 2022). This reductionist framework is essential for establishing cause–effect relationships before advancing to more complex soil systems. Despite previous research, several critical knowledge gaps persist: first, the temporal dynamics of Fe-plaque formation and its effectiveness across acute to chronic As exposure remain poorly resolved; second, the interplay between Fe amendment, root anatomical adaptation, and ROL regulation under prolonged As stress is inadequately understood; and third, the potential trade-offs between As sequestration and the metabolic costs associated with Fe-plaque maintenance require systematic evaluation.

This study employs a multi-temporal hydroponic experiment to investigate Fe-mediated mitigation of As toxicity in rice seedlings, with a specific focus on root morphological plasticity and ROL patterns. We hypothesize that Fe amendment differentially alleviates As-induced physiological suppression across exposure durations and concentrations through enhanced plaque formation and subsequent root anatomical modifications. By integrating quantitative assessments of Fe-plaque development, root porosity, aerenchyma formation, and ROL kinetics, we aim to: (1) delineate the temporal efficacy of Fe in counteracting As toxicity, (2) identify the root morphological and anatomical traits most responsive to Fe supplementation, and (3) establish the relationship between plaque properties and As sequestration capacity. Our findings will advance the mechanistic understanding of plant adaptive responses to metalloid stress and inform the development of targeted Fe-based remediation strategies for As-contaminated wetland ecosystems.

## 2 Materials and methods

### 2.1 Experimental setup

In this study, we used rice seeds (*Oryza sativa* L. Jiafuzhan) obtained from the School of Life Sciences, Xiamen University, China. To prepare the seeds for experimental treatments, the following streamlined protocol was followed. Step 1, seed selection and sterilization: Fully matured rice seeds were selected to ensure consistent germination and growth. The seeds were first rinsed with ultrapure water to remove surface contaminants, then disinfected by immersion in 70% ethanol for 30 s. Subsequently, the seeds were soaked in 10% hydrogen peroxide (H_2_O_2_) for 10 min for enhanced sterilization (Miche and Balandreau, 2001). Finally, the seeds were rinsed with sterile ultrapure water to remove residual disinfectants. Step 2, seed germination: Sterilized seeds were placed in an illumination incubator (Ningbo Saifu, China) at 25 °C in complete darkness for 24 h to promote water absorption and initiate germination, facilitating uniform sprout development. Step 3, seedling establishment: Post-germination, seeds were evenly distributed on a sand net to support root development. Irrigation commenced with Hoagland solution (details in **Supplementary Table S2**) at 25% concentration. The nutrient solution was replaced every 3 days, with the concentration gradually increased until full-strength (100%) Hoagland solution was attained (Majidi et al., 2025; Mei et al., 2021). Step 4, seedling growth and preparation: Seedlings were cultivated in a greenhouse for 1 month under controlled conditions (25 ± 2°C with a 12-h light/12-h dark photoperiod) (Cui et al., 2023). Following cultivation, seedlings exhibiting uniform growth were selected for subsequent experimental treatments.

### 2.2 Treatment of pot-cultivated seedlings

After transferring seedlings into hydroponic conditions, 1-month seedlings were subjected to varying concentrations of sodium arsenite (NaAsO_2_) and ferrous sulfate (FeSO_4_) to assess their combined effects over different exposure periods according to previous studies (Mei et al., 2021; Wang et al., 2021). The experimental design included three arsenite levels (Control: 0 μmol/L; Low As: 10 μmol/L; High As: 30 μmol/L) and amended ferrous iron concentrations (No additional Fe: 0 μmol/L; With additional Fe: 100 μmol/L). The following treatments were applied:

(1) CK+No-Fe: Control + No additional Fe; (4) CK+With-Fe: Control + Fe addition;

(2) LAs+No-Fe: Low As + No additional Fe; (5) LAs+With-Fe: Low As + Fe addition;

(3) HAs+No-Fe: High As + No additional Fe; (6) HAs+With-Fe: High As + Fe addition.

The study examined rice seedlings during the vegetative growth stage, prior to the booting phase. Seedlings underwent As exposures for 1 day, 1 week, and 1 month, with three replicates per treatment. All pots were randomly arranged in the illumination incubator under the same conditions described above.

### 2.3 Plant growth attributes and root morphology

To assess seedling growth, fresh weight biomass was measured using an electronic balance after collecting uniformly grown seedlings from each replicate. The dry weight of the seedlings was determined after oven-drying at 60 °C for 72 h until a constant weight was achieved. Fully expanded true leaves on plants were collected in triplicate for chlorophyll pigment analysis. Fresh leaves (~0.2 g) were ground, homogenized, and extracted using 90% acetone in darkness at 4 °C for 24 h. The extract was then centrifuged at 3000 rpm for 15 min, and the chlorophyll content in the supernatant was quantified using a UV–Vis spectrometer (Beijing Ruili, China) at wavelengths of 663 and 645 nm. Root morphology—including root length, diameter, surface area, and volume—was analyzed using a scanning apparatus (LA-S Plant Image Analyzer, Hangzhou Wseen, China).

To investigate the effects of As and Fe on plant root lignification, transverse root sections were prepared and analyzed. Fresh root segments (6–8 cm) were hand-sectioned using a rotary microtome. The sections were mordanted with ferric ammonium sulfate and stained with diluted hematoxylin to visualize lignified cell walls (Ji et al., 2006). Stained sections were subsequently examined and imaged under a photomicroscope.

### 2.4 Root porosity determination

Root porosity (POR) was determined using the pycnometer method. Fresh lateral roots (0.5 g) were cut into segments and wiped with air-laid paper prior to measurement (Mei et al., 2014). POR was calculated using Equation 1:

(1) POR(%)=100×(PWVR-PWR)/(PW+FR-PWR)

where *PWVR* (g) is the weight of the pycnometer with ultrapure water and vacuumed root; *PWR* (g) is the weight of the pycnometer with ultrapure water and fresh root; *PW* (g) is the weight of the pycnometer filled with ultrapure water; and *FR* (g) is the weight of the fresh root.

### 2.5 Assessment of radial oxygen loss rates

The rate of ROL in seedlings was measured using a modified titanium (Ti^3+^) citrate method (Mei et al., 2021, Mei et al., 2014). Roots were immersed in a deoxygenated titanium (Ti^3+^) citrate solution under dark conditions and sealed with a 2 cm paraffin oil layer to prevent atmospheric oxygen intrusion. The incubation was conducted for 24 h at room temperature under a 12-h light/12-h dark cycle. Afterward, the solution was gently agitated and sampled with syringes. The absorbance of the oxidized titanium (Ti^3+^) citrate was measured at 527 nm using a UV–Vis spectrometer (Beijing Ruili, China), corresponding to the absorption peak of its oxidized form. The ROL rate (mM O_2_ day^−1^ g^−1^ DW) was calculated using Equation 2:

(2) $$\text{ROL rate }=\;(C_{i}\;-\;C_{b})\;\times \;V/W$$

where *V* is the initial volume (L) of Ti^3+^-citrate; *C_b_* is Ti^3+^ concentration (μmol/L) in the blank; *C_i_* is the Ti³^+^ concentration (μmol/L) after 24 h incubation with the rice seedling; and *W* is the root dry weight (g DW).

### 2.6 Root iron plaque extraction

Iron plaque was extracted from fresh roots with the dithionite–citrate–bicarbonate (DCB) solution, following established protocols to determine As and Fe contents in the plaque (Mei et al., 2021, Mei et al., 2014). The iron plaque extract was digested with H_2_SO_4_/H_2_O_2_ at 120°C for 6 h. The digested mixture was then centrifuged at 8000 rpm for 5 min (Hettich Universal 320R Centrifuge, Germany) and filtered through a 0.45 μm membrane. Fe concentration was measured via atomic absorption spectroscopy (AAS Vario 6, Thermo Fisher Scientific, USA), and As concentration was assessed by atomic fluorescence spectrometry (AFS-930, Beijing Jitian, China).

### 2.7 Fe and As translocation into the seedlings

Roots (without iron plaque), stems, and leaves of all treated seedlings were freeze-dried at –20°C, weighed to determine dry weight and biomass, and analyzed for Fe and As content. Each plant part was ground using a ball mill and passed through an 80-mesh sieve to obtain a fine powder. Subsamples (~0.2 g) were digested overnight with 5 mL nitric acid at room temperature. The following day, 2 mL hydrogen peroxide was added and heated at 120 °C for 6 h until fully digested (Mei et al., 2021). The cooled digest solution was then diluted with 5% hydrochloric acid and stored at –20 °C for subsequent analysis. Fe and As concentrations were determined as described above.

The translocation factor (TFAs), defined as the ratio of As content in aerial parts (leaf and stem) to that in roots, was calculated using Equation 3:

(3) $$\text{TFAs }=\;100\%\;\times \;Aerial_{As}\;/Root_{As}$$

where Aerail_As_ is the As content in the leaf and stem, *Root_As_* is the As content in the root.

### 2.8 Statistical analysis

The significance of measured parameters was determined using one-way ANOVA. Differences among treatments were further analyzed with one-way ANOVA followed by Duncan’s multiple comparison test. Geisser–Greenhouse correction was applied to account for variance homogeneity. Different letters indicated significant differences among treatments at *p* < 0.05. Spearman’s correlation analysis was used to identify relationships among parameters. Redundancy analysis (RDA) was performed to explore the relationships between plant morphological variables and Fe/As distribution using R with the Vegan package. Data statistics and visualizations were performed using SPSS Statistics 23.0, GraphPad Prism 9, and the R statistics package (version 4.0.2, https://www.r-project.org).

## 3 Results

### 3.1 Chlorophyll content under arsenic stress

**Figure 1:**
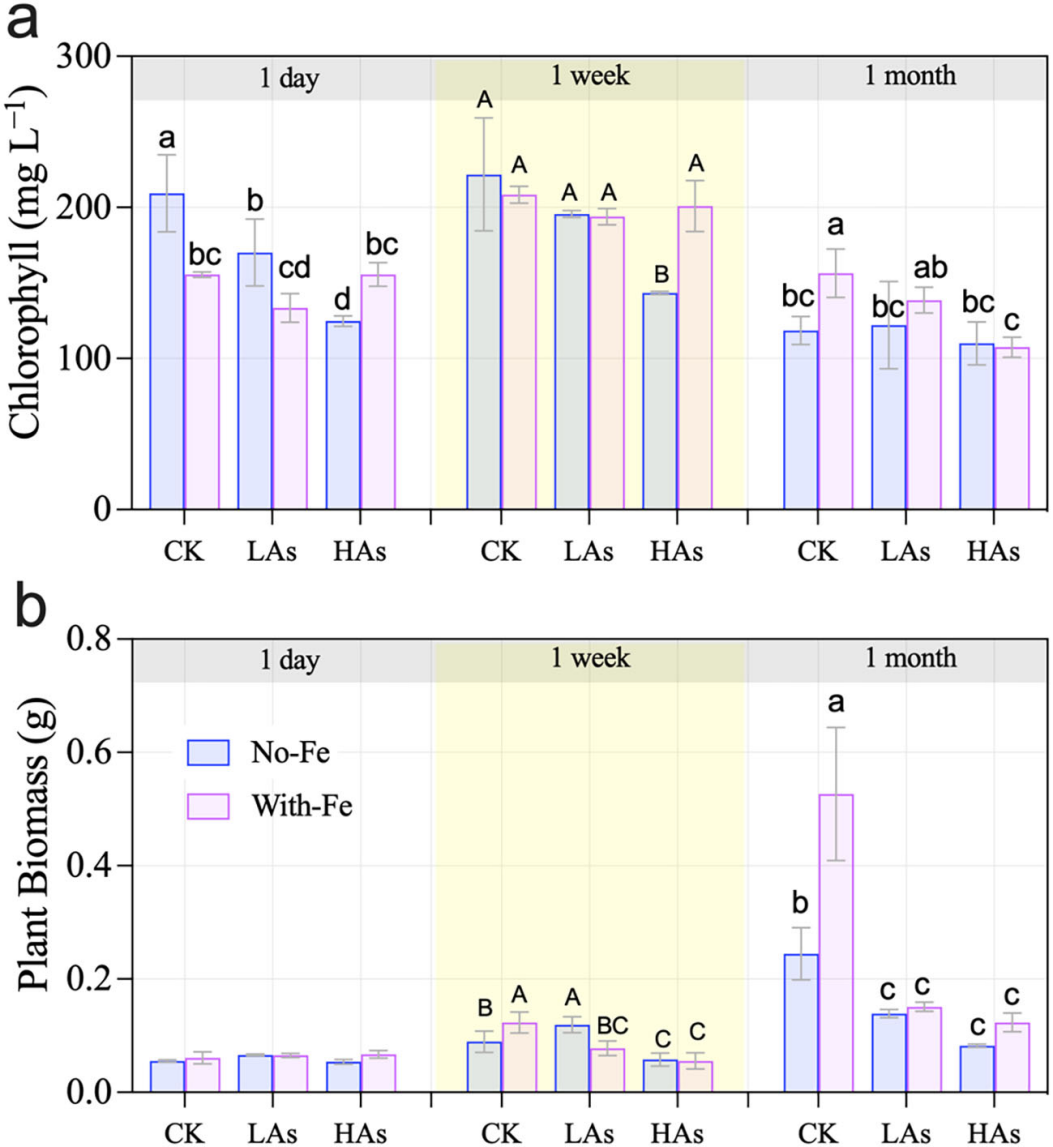
Chlorophyll content **(a)** and biomass **(b)** of the rice seedlings *(O. Sativa)* exposed to different As and Fe treatments after transplanting 30-day old seedlings. Different letters within each time group indicate significant differences among Fe and As treatments at *p<* 0.05 according to Duncan’s test.

As exposure significantly influenced chlorophyll content in rice seedlings, with both exposure duration and concentration playing critical roles. Chlorophyll levels exhibited an initial increase followed by a decrease over time, peaking at 263.84 mg·L⁻¹ after 1 week of treatment across all groups (**Figure 1a**). Although As exposure significantly affected chlorophyll content, plants under prolonged (1-month) low-level As (LAs) stress showed partial recovery, with chlorophyll content slightly exceeding that of the control group (CK), although this difference was not statistically significant (*p* > 0.05; **Figure 1a**). Importantly, Fe supplementation demonstrated a time-dependent mitigation effect. Although it did not alleviate As-induced chlorophyll loss after 1 month, high-concentration Fe application significantly enhanced chlorophyll content during the early stress phase (1 week; *p <* 0.05; **Table 1**).

**Table 1:** Effects of As and Fe interactions on root morphology-related parameters in *O. Sativa* seedlings (n=3).

Time	Fe amended	As treatment	Root length (cm)	Root surface (cm^2^)	Root volume (cm^3^)	Root diameter (mm)	Porosity of root (%)	Root dry weight (g DW)
1 day	No-Fe	CK	95.9 ± 10.4a	5.52 ± 1.28b	0.03 ± 0.01b	0.17 ± 0.01c	4.72 ± 0.70a	0.011 ± 0.001b
		LAs	114.7 ± 20.6a	7.76 ± 1.65a	0.05 ± 0.01a	0.20 ± 0.01ab	4.59 ± 1.01ab	0.012 ± 0.001ab
		HAs	95.2 ± 3.6a	6.32 ± 0.61ab	0.04 ± 0.01ab	0.20 ± 0.01ab	3.71 ± 0.96ab	0.012 ± 0.001ab
	With-Fe	CK	112.7 ± 17.0a	6.88 ± 1.42ab	0.04 ± 0.01ab	0.17 ± 0.02c	3.65 ± 1.09ab	0.02 ± 0.004a
		LAs	98.1 ± 2.03a	6.91 ± 0.53ab	0.05 ± 0.01a	0.21 ± 0.01a	3.66 ± 0.17ab	0.02 ± 0.001a
		HAs	105.1 ± 10.7a	7.05 ± 0.69ab	0.05 ± 0.004a	0.18 ± 0.01bc	3.10 ± 0.35b	0.02 ± 0.002a
	**Fe addition**		**NS**	**NS**	***p* = 0.020**	***p* = 0.003**	***p* = 0.012**	***p* = 0.01**
1 week	No-Fe	CK	102.3 ± 12.6C	9.63 ± 1.15B	0.10 ± 0.01B	0.40 ± 0.09A	6.30 ± 0.19A	0.01 ± 0.002B
		LAs	196.5 ± 25.5AB	25.7 ± 3.99A	0.33 ± 0.09A	0.41 ± 0.05A	4.85 ± 0.10B	0.02 ± 0.001A
		HAs	145.5 ± 6.9BC	15.89 ± 2.05B	0.17 ± 0.04B	0.35 ± 0.01A	6.71 ± 0.57A	0.01 ± 0.001B
	With-Fe	CK	148.4 ± 8.9BC	14.61 ± 2.58B	0.16 ± 0.05B	0.35 ± 0.02A	3.34 ± 0.73C	0.02 ± 0.004A
		LAs	212.2 ± 10.6A	33.0 ± 11.66A	0.42 ± 0.13A	0.37 ± 0.03A	3.68 ± 0.57BC	0.02 ± 0.001A
		HAs	243 ± 22.7A	27.78 ± 2.48A	0.33 ± 0.05A	0.34 ± 0.01A	3.69 ± 1.13BC	0.01 ± 0.001B
	**Fe addition**		**NS**	**NS**	***p* = 0.002**	***p* = 0.001**	***p* = 0.001**	**NS**
1 month	No-Fe	CK	319.7 ± 117.3a	40.54 ± 13.65b	0.60 ± 0.25b	0.41 ± 0.04bc	5.75 ± 0.77b	0.04 ± 0.01a
		LAs	268.2 ± 41.6a	31.03 ± 2.51b	0.52 ± 0.21b	0.38 ± 0.01cd	4.42 ± 0.65bc	0.04 ± 0.02a
		HAs	427.5 ± 63.4a	43.77 ± 9.24b	0.52 ± 0.13b	0.34 ± 0.01d	9.13 ± 0.72a	0.04 ± 0.03a
	With-Fe	CK	409.5 ± 128.2a	68.87 ± 20.92a	1.19 ± 0.40a	0.48 ± 0.02a	9.44 ± 0.35a	0.06 ± 0.01a
		LAs	312.4 ± 55.2a	37.79 ± 5.68b	0.53 ± 0.03b	0.40 ± 0.02bc	4.00 ± 1.74c	0.04 ± 0.004a
		HAs	506.2 ± 128.7a	67.41 ± 22.62a	1.00 ± 0.42ab	0.44 ± 0.04ab	3.85 ± 0.68c	0.04 ± 0.003a
	**Fe addition**		**NS**	**NS**	***p* = 0.031**	***p* = 0.002**	***p* = 0.021**	**NS**
**Time treatment**			***p* < 0.001**	***p* < 0.001**	***p* < 0.001**	***p* < 0.001**	***p* < 0.001**	***p* < 0.001**

Different letters in each time group indicate significant differences among Fe and As treatments at *p* < 0.05 by Duncan’s test. Bold values indicate significant differences for each parameter with regard to the Fe addition treatment or the time treatment.

### 3.2 Biomass responses to arsenic–iron interactions

As exposure significantly inhibited rice seedling growth, with root dry weight showing a clear time- and concentration-dependent response (**Figure 1b**). Although root biomass generally increased over the 1-month experimental period, reaching a maximum of 0.52 g in the control groups, it was substantially suppressed under As stress. After 1 month of exposure, root dry weight showed significant concentration-dependent inhibition (CK > LAs, HAs), declining to as low as 0.12 g in the HAs treatment. Notably, Fe supplementation effectively mitigated this suppression, particularly under prolonged stress. After 1 month, Fe-amended treatments produced plant biomass that was 1.18–1.28 times greater than their respective no-additional-Fe counterparts at equivalent As contents, demonstrating Fe’s crucial role in maintaining root growth under chronic As toxicity.

### 3.3 Dynamics of root morphology and dry weight

#### 3.3.1 Temporal changes in root architecture

Root morphological parameters (length, surface area, volume, and average diameter) increased significantly during the 1-month cultivation period, peaking after 1 month of Fe amendment (root length: 409.5 cm; surface area: 68.87 cm²; volume: 1.19 cm³; diameter: 0.48 mm; *p* < 0.01; **Table 1** and **Supplementary Figure S1**). The response patterns were shaped by the combined effects of As concentration and exposure duration. LAs exposure after 1 week stimulated root growth (LAs > HAs > CK), whereas 1-month HAs exposure suppressed root volume and diameter (CK > HAs > LAs). Notably, under prolonged 1-month As stress, root length and surface area followed the order HAs > CK > LAs (**Table 1**; *p* > 0.05). Fe supplementation significantly alleviated the As-induced suppression of root volume and diameter after 1 month of exposure (**Table 1**).

#### 3.3.2 Root dry weight

The RDW results exhibited distinct temporal and concentration-dependent responses to As exposure (**Table 1** and **Supplementary Figure S1**). Although RDW increased over time across all treatments, reaching a maximum of 0.07 g in the control groups after 1 month, As exposure significantly altered this progression. After 1 week, low As (LAs) treatment induced a slight stimulatory effect on RDW (LAs > CK > HAs; *p* > 0.05). In contrast, prolonged exposure (1 month) resulted in significant concentration-dependent suppression, with RDW following a decreasing order of CK > LAs (**Supplementary Figure S1**).

Notably, Fe amendment effectively alleviated this chronic As toxicity, enhancing RDW by 1.18–2.39 times compared with the corresponding non-Fe treatments at equivalent As levels after 1 month.

### 3.4 Root porosity and aerenchyma formation

Root porosity increased in a time-dependent manner under As–Fe interactions, peaking at 9.84% after 1 month of treatment (**Table 1**). Acute As exposure (1 day) suppressed porosity (CK > LAs > HAs), whereas prolonged HAs exposure (1 week and 1 month) promoted it (HAs > CK > LAs). Counterintuitively, Fe amendment reduced porosity, with No-Fe groups exhibiting 1.32–1.91 times higher porosity than their Fe-treated counterparts at 1 week (*p* < 0.05).

**Figure 2:**
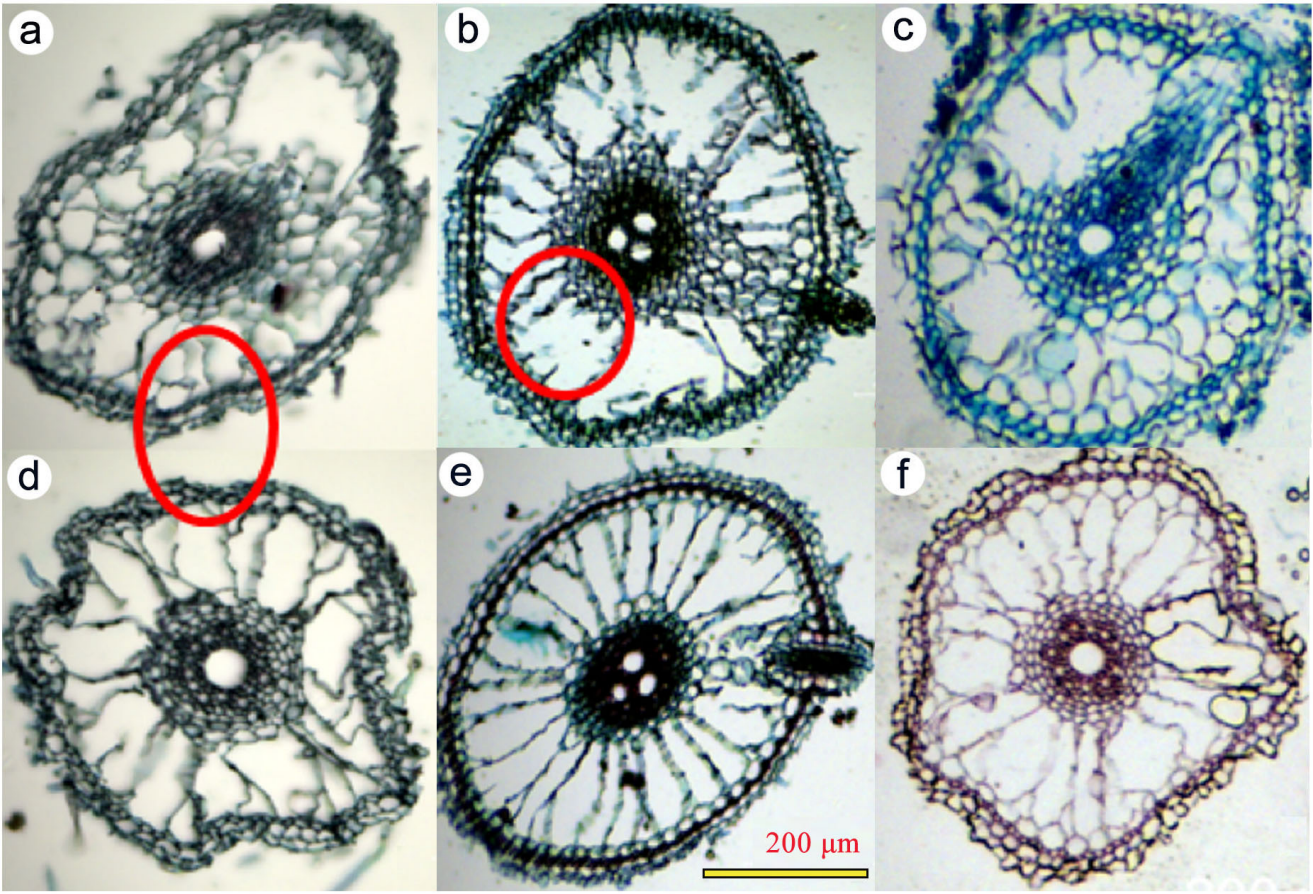
Anatomical microscopy images of rice root transverse sections exposed to As and Fe treatments for 1 month at the tillering stage. **(a)** Control without Fe; **(b)** low As without Fe_;_**(c)** high As without Fe; **(d)** control with Fe amendment; **(e)** low As with Fe amendment; and **(f)** high As with Fe amendment.

Anatomically, aerenchyma formation initiated within 1 day (accounting for ~17% of the root cross-sectional area) and progressed to 46.3% under 1-month As–Fe co-treatment. The aerenchyma displayed radial, wheel-like structures (**Figure 2**) formed via lysigenous programmed cell death (PCD) of cortical parenchyma. Iron supplementation tended to increase the proportion of gas-filled tissue.

### 3.5 Radial oxygen loss (ROL) and iron plaque dynamics

**Figure 3:**
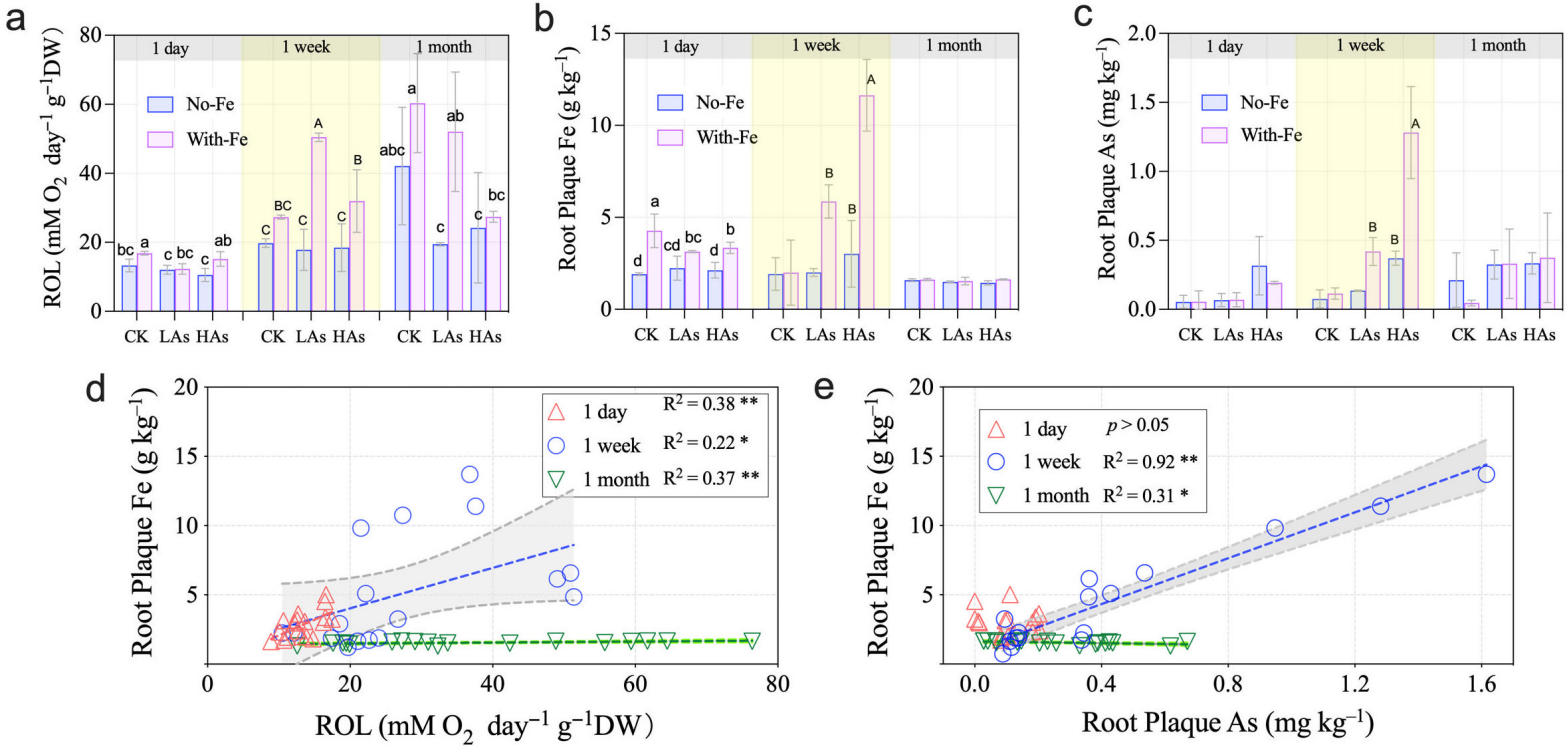
Root exudation of radial oxygen loss (ROL) and root plaque–bound metals in *Oryza sativa* exposed to As and Fe treatments. **(a)** ROL rates; **(b)** root plaque Fe; **(c)** root plaque As; **(d)** ROL vs. plaque Fe; and **(e)** plaque As vs. plaque Fe. Different letters within each time group indicate significant differences among treatments at *p <* 0.05 according to Duncan’s test.

The ROL rate increased significantly with prolonged treatment duration, reaching 64.46 mM O_2_·day⁻¹·g⁻¹ DW at 1 month (**Figure 3**). After 1-week treatment, the As content in the iron plaque on the root surface showed a significant positive correlation with the Fe content (R^2^ = 0.92, *p* < 0.05). After 1 month, the Fe plaque content did not increase significantly (**Figure 3e**), yet As accumulation continued to rise.

Overall, ROL intensity increased with root growth in conjunction with iron plaque development (**Figures 3a, d**). As content negatively regulated ROL (CK > LAs > HAs), with HAs treatment suppressing the ROL rate to 50% of the control level after 1 month. Fe supplementation counteracted this As-induced inhibition, elevating ROL rates by 1.38–2.83 times in As-treated groups at 1 week.

### 3.6 Arsenic translocation and mobility

**Figure 4:**
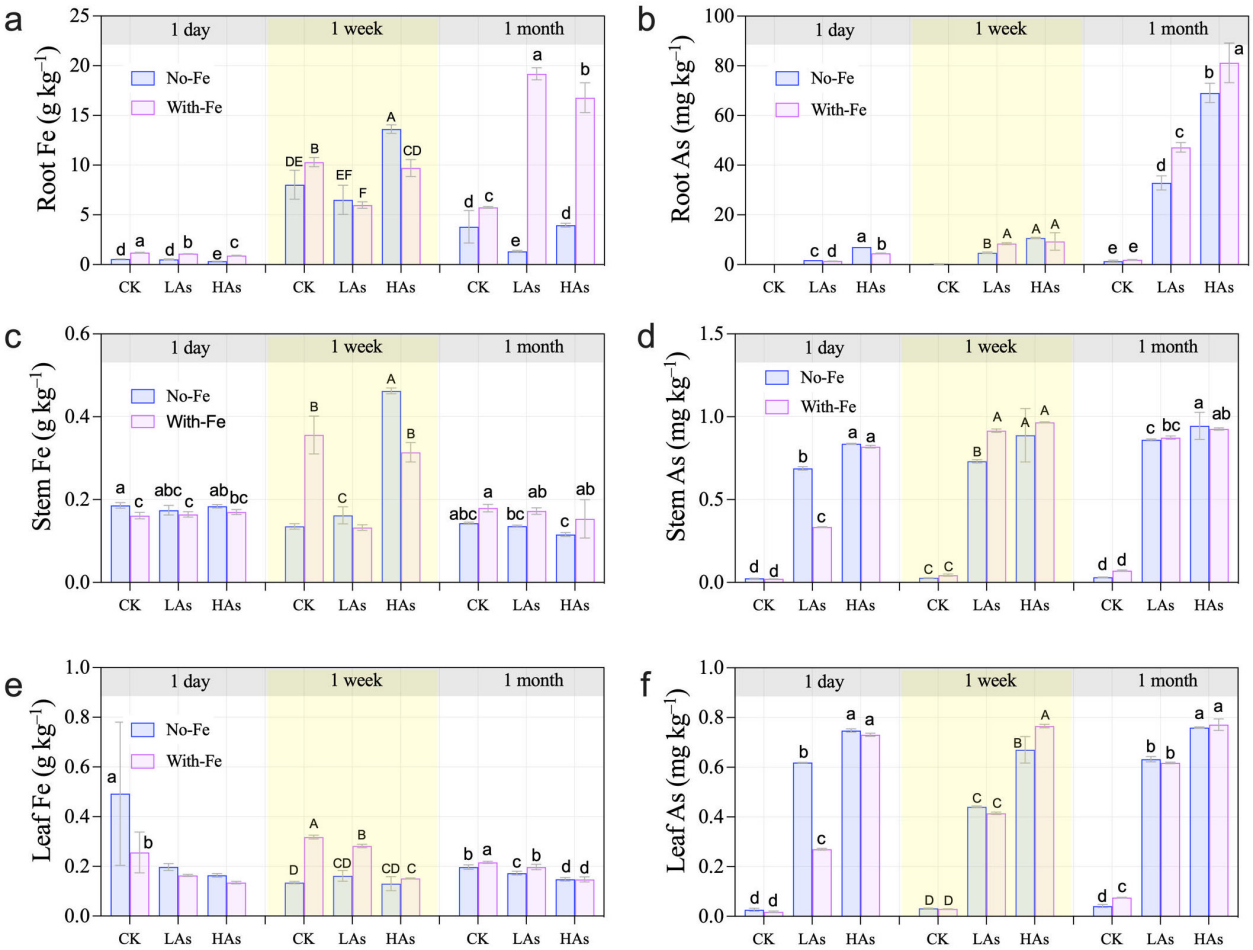
Content of As and Fe in plant tissues of *Oryza sativa* under As and Fe treatments. **(a)** Fe content in the root; **(b)** As content in the root; **(c)** Fe content in the stem; **(d)** As content in the stem; **(e)** Fe content in the leaf; and **(f)** As content in the leaf. Different letters within each time group indicate significant differences among treatments at *p* < 0.05 according to Duncan’s test.

The contents of As and Fe in plant tissues of *O. sativa* are shown in **Figure 4**. Fe treatment significantly affected Fe contents in roots, stems, and leaves, while exposure duration markedly influenced the redistribution of As and Fe within plant tissues (**Supplementary Table S1**). After 1 month of treatment, the Fe-treated group showed significantly higher root As content than the non-Fe-treated group under the same As exposure (**Figure 4b**).

As exposure for 1 week resulted in higher arsenic translocation factors (TF_As_), with values of 0.22–0.73 at 1 day and 0.37–0.77 at 1 week. In contrast, 1-month treatment drastically reduced TF_As_ to 0.02–0.05 (**Supplementary Figure S2**). Notably, TF_As_ under 1-week As–Fe co-treatment were 36.5 times higher than under 1-month exposure, indicating a sharp decline in As translocation efficiency over time. As content inversely regulated TF_As_ during acute exposure (1 day: LAs > HAs) but exhibited the opposite trend at 1 week (HAs > LAs).

**Figure 5:**
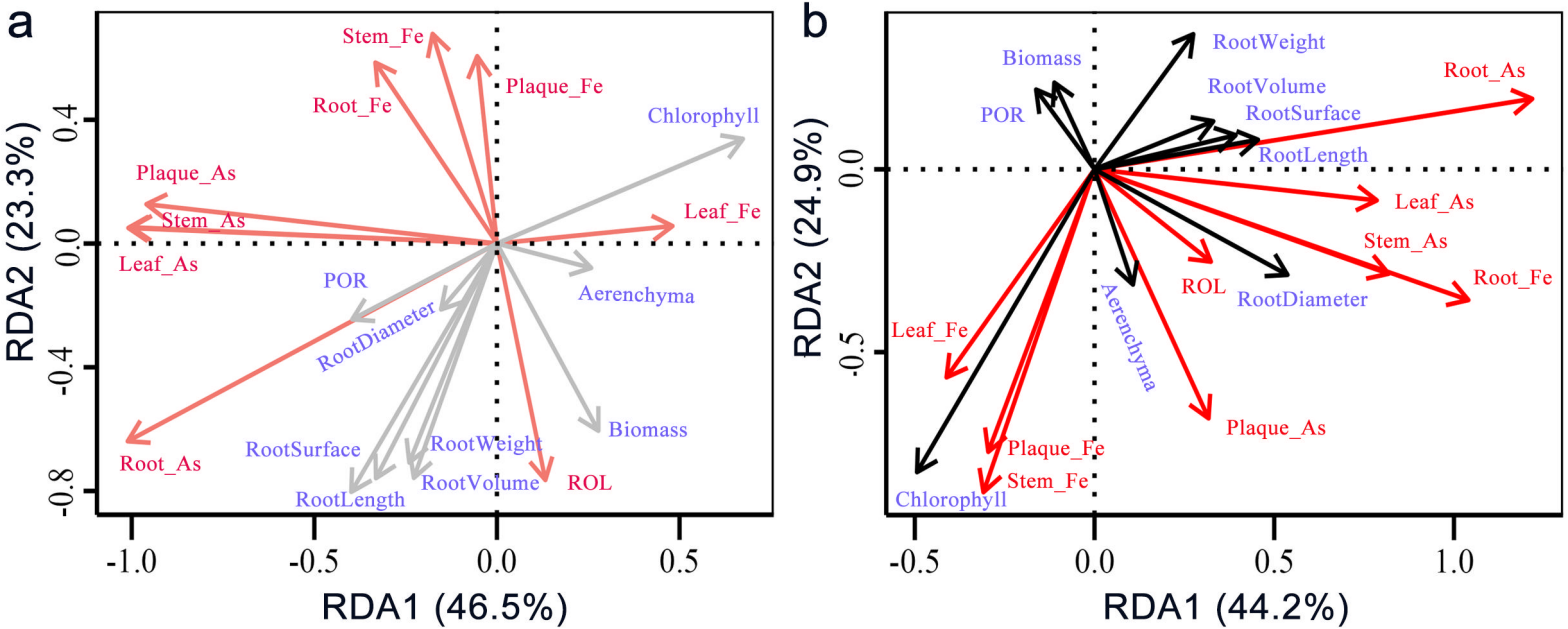
Redundancy analysis (RDA) between plant morphological traits and As and Fe parameters. **(a)***Oryza sativa* under As treatment without Fe; and **(b)***Oryza sativa* under As treatment with Fe amendment.

**Figure 6:**
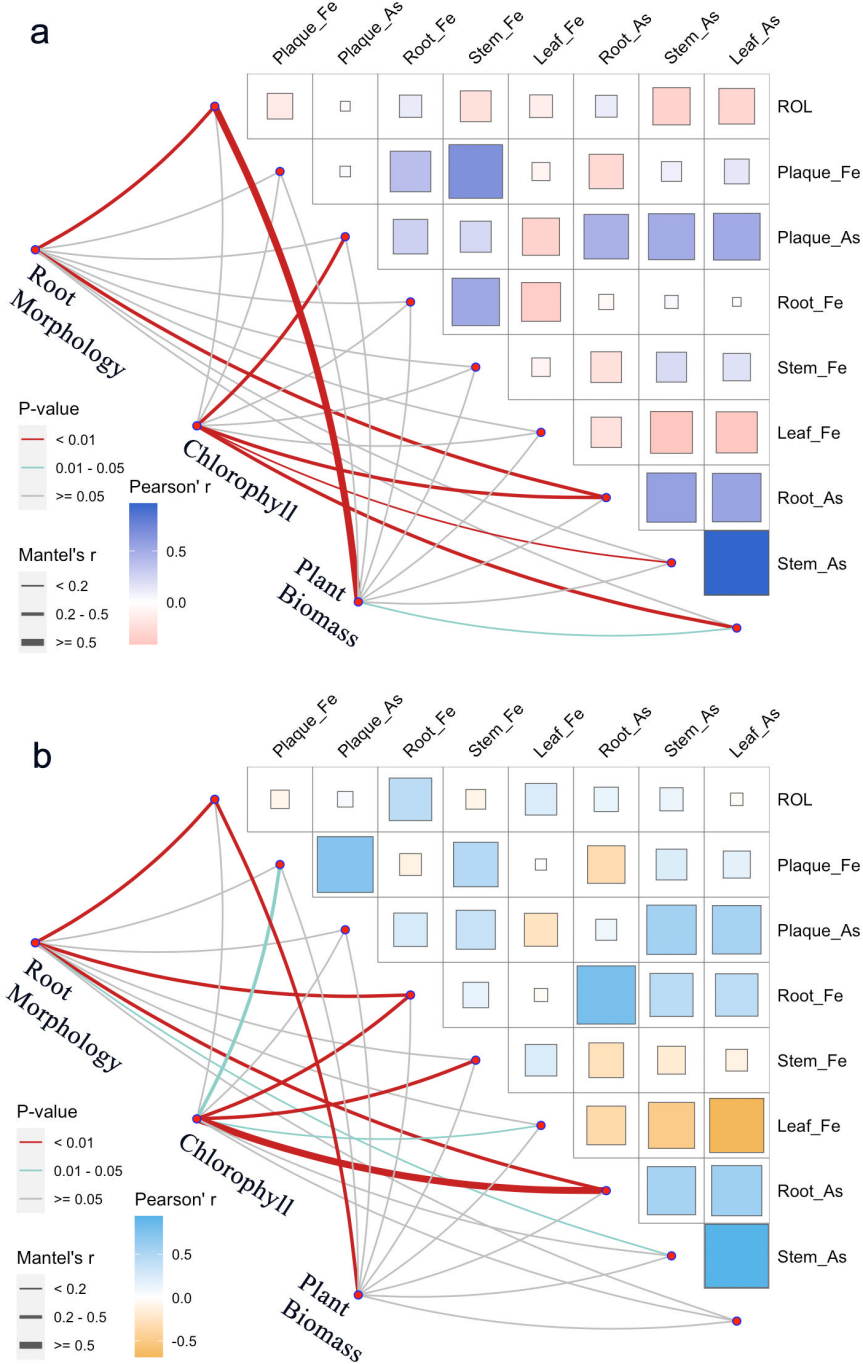
Mantel test and correlation matrix among plant morphological indices and Fe and As parameters. **(a)***Oryza sativa* under As treatment without Fe; and **(b)***Oryza sativa* under As treatment with Fe amendment.

Fe amendment significantly altered As translocation patterns. Acute Fe supplementation significantly reduced TF_As_ by 38% (*p* < 0.001), indicating enhanced root sequestration via Fe-plaque formation. Meanwhile, Fe treatment increased As uptake by the roots, as the iron plaque acted as a sustained sink for As enrichment (**Figure 4b**). Root As content was positively correlated with root morphogenesis and chlorophyll content (**Figure 5**), suggesting that As stress promoted root architectural development. In Fe-treated groups, both root morphogenesis and chlorophyll content were significantly positively correlated with root Fe content (**Figure 6**), demonstrating that Fe amendment enhances the detoxification capacity of rice seedlings.

## 4 Discussion

### 4.1 Temporal dynamics of arsenic phytotoxicity and iron-mediated mitigation

Our results demonstrate a biphasic response of chlorophyll content to As stress: an initial significant reduction under 1-week exposure, followed by a compensatory recovery under prolonged low-level As. This pattern suggests the activation of dynamic detoxification mechanisms, such as phytochelatin synthesis and antioxidant defenses, over time (Gaikwad et al., 2020; Mondal et al., 2022). The mitigation by Fe was both time- and concentration-dependent. Notably, because the baseline Hoagland solution contains Fe³^+^, all plants had access to Fe, enabling some plaque formation even without external Fe supplementation. As a result, the Fe and As contents in the iron plaque showed a significant positive correlation after both 1-week and 1-month exposure periods across all treatments (**Figure 3e**). The pronounced efficacy of high-dose Fe after a 1-week incubation underscores the role of rapid Fe-plaque formation in physically sequestering As at the root surface during the acute stress phase (Mei et al., 2021; Zulfiqar and Ashraf, 2022). Under prolonged exposure, sustained Fe supply likely served as a nutrient reservoir supporting ongoing chlorophyll synthesis and contributing to the observed recovery, as evidenced by differences between the With-Fe and No-Fe control groups.

The persistent biomass suppression under 1-month As exposure reflects the cumulative metabolic cost of detoxification. The modest biomass improvement with Fe supplementation—despite a more pronounced recovery in chlorophyll—implies a strategic reallocation of energy (Gaikwad et al., 2020). In this scenario, resources are prioritized for essential survival processes (e.g., maintaining detoxification pathways and antioxidant systems) over growth (Liu et al., 2011; Mei et al., 2012), a trade-off characteristic of metallophytes (Farooq et al., 2016; Maestri et al., 2010; Zulfiqar and Ashraf, 2022). This highlights the temporal complexity of As–Fe interactions: although Fe can rapidly mitigate As uptake and acutely protect photosynthesis, long-term growth benefits remain limited by the underlying metabolic burden associated with chronic stress.

### 4.2 Arsenic-driven root plasticity and iron’s selective protection

Arsenic As content and exposure duration jointly shaped distinct root morphological patterns. The initial stimulatory effect of low As (LAs) on root growth aligns with the hormesis phenomenon, whereas chronic exposure led to suppression, particularly in root volume and diameter. The observed elongation of roots under 1-month HAs stress (HAs > LAs > CK for length/surface area) represents a compensatory adaptation to enhance nutrient foraging in a toxic environment (Gaikwad et al., 2020; Hossain et al., 2007), consistent with strategies reported in metallophytes (Eissenstat, 2000; Sobrino-Plata et al., 2013).

Fe amendment played a selective protective role, significantly restoring root volume and diameter but not influencing length and surface area. This selectivity likely stems from the mechanism of Fe-plaque formation, which immobilizes As in the root apoplast, thereby reducing its cytotoxicity and preserving the cellular integrity necessary for radial expansion (Paulelli et al., 2019). This hierarchical prioritization in root development underscores the plant’s sophisticated strategy for resource allocation under metal stress.

### 4.3 Aerenchyma formation, porosity, and their regulation by As-Fe interaction

The treatment groups at different time points showed significant differences in root morphology-related parameters under the LSD test (*p* < 0.001; **Table 1**). The time-dependent increase in root porosity and aerenchyma formation under As stress indicates an adaptive response to potential hypoxia and oxidative stress. The counterintuitive reduction in porosity by Fe amendment suggests that Fe-plaque may physically impede gas diffusion or alter hormonal signals (e.g., ethylene) governing aerenchyma formation (Cheng et al., 2015; Rogers et al., 1992; Zhang et al., 1998).

The partial reversal of As-induced aerenchyma suppression by Fe is a critical finding (Cheng et al., 2015; He et al., 2004; Parsons et al., 2013). It correlates with Fe-plaque-mediated As sequestration, which may reduce As-triggered disruption of lysigenous programmed cell death (PCD) in the cortex (Robinson et al., 2003). The formation of radial, wheel-like aerenchyma structures highlights a tightly regulated anatomical adaptation. The interplay among As toxicity, Fe remediation, and aerenchyma development reflects the competing demands of oxygen transport (via aerenchyma) and detoxification (via Fe-plaque).

### 4.4 The interplay of ROL, iron plaque, and arsenic sequestration

Redundancy analysis (RDA) revealed that plaque Fe content was negatively correlated with ROL in the control group, whereas under Fe amendment, both root Fe and As contents showed positive correlations with ROL (**Figure 5**). The negative correlation between plaque Fe and ROL in controls suggests that natural Fe deposition can slightly impede oxygen diffusion. Under As stress, however, Fe amendment reversed the As-induced suppression of ROL and correlated positively with both root Fe and As content (Mei et al., 2012). This shift indicates that Fe-plaque not only immobilizes As but also helps maintain a more oxidized rhizosphere, potentially mitigating the reductive mobilization of As(III). The observed decoupling of ROL from root porosity—contrary to some ROL models ROL (Mei et al., 2014), emphasizes the dominant role of fine, non-aerenchymatous lateral roots in oxygen release in rice (Cheng et al., 2010; Mei et al., 2012), and underscores that Fe’s primary mitigation function operates through chemical immobilization rather than through alterations in root anatomical traits (Abedin et al., 2002; Hu et al., 2019; Rebouillat et al., 2009).

### 4.5 Mechanisms of reduced arsenic translocation and enhanced detoxification

The drastic decline in the arsenic translocation factor (TF_As_) over time, particularly under Fe amendment, underscores a fundamental shift in plant strategy—from initial As distribution to robust root sequestration over the 1-month period. This process is initiated by the formation of Fe plaque formation (Liu et al., 2011), which acts as a primary barrier to reduce As accumulation within the root, as confirmed by the sharp reduction in TFAs following acute Fe treatment. However, Fe amendment increased As accumulation in the roots under prolonged stress, which may be attributed to Fe-plaque functioning as a sustained sink for As enrichment or to Fe-induced physiological changes that enhanced As immobilization (Mei et al., 2014).

In Fe-treated groups, both root morphogenesis and chlorophyll content were significantly positively correlated with root Fe content (**Figure 6b**). Fe amendment orchestrates a multifaceted protective response. Systemically, it enables the maintenance of higher chlorophyll content, revealing a direct positive correlation with Fe treatment that underscores the protection of the photosynthetic machinery (Wu et al., 2022; Zhao et al., 2010). At the root level, anatomical adaptations also occur. Root porosity showed an inverse correlation with As stress, suggesting structural modification toward a more resilient phenotype. PCA analysis indicated that root morphogenesis, ROL, and POR were major contributors to Principal Component 1 (PC1, **Table 2**).

**Table 2:** PCA results of root morphology and biological indices of *O. sativa* under As and Fe treatments (n=54).

Index	PC1	PC2	Communalities
Root length	**0.923**	−0.161	0.879
Root surface	**0.959**	−0.052	0.923
Root volume	**0.960**	−0.005	0.921
Biomass	**0.861**	0.197	0.781
Root weight	**0.844**	−0.359	0.842
Root diameter	**0.778**	0.278	0.683
ROL	**0.708**	0.169	0.529
POR	**0.570**	−0.036	0.327
Aerenchyma	0.487	**0.551**	0.541
Chlorophyll	-0.268	**0.771**	0.667

Note: Bold values represent the indicators of principal component components in confidence intervals.

**Figure 7:**
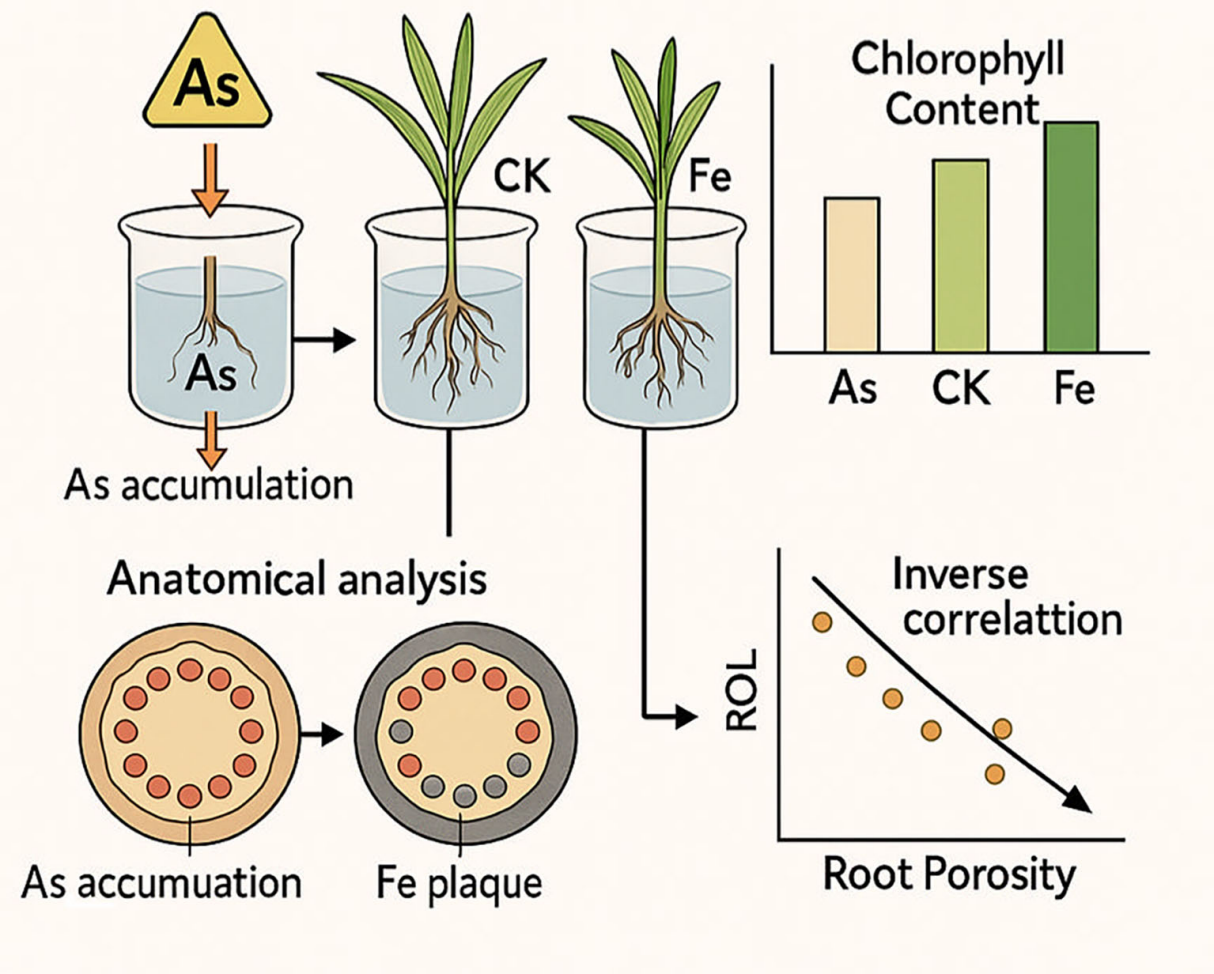
Proposed mechanistic model of iron-mediated mitigation of arsenic toxicity in rice roots.

These interconnected mechanisms (as shown in **Figure 7**)—rhizosphere sequestration, physiological maintenance, and anatomical adaptation—synergistically contribute to the observed improvement in biomass under Fe supplementation. This stands in contrast to the pronounced growth suppression in As-stressed groups without Fe. Thus, Fe amendment does not merely immobilize As passively; it fosters a more robust physiological state by protecting root meristems and photosynthetic centers, enhancing the intrinsic detoxification capacity of rice seedlings, and ultimately ensuring better growth under stress.

## 5 Conclusions

This study elucidates the temporal dynamics and mechanistic interplay of arsenic–iron interactions in modulating root morphogenesis and radial oxygen loss (ROL) in rice seedlings. Arsenic exposure for 1 week induced acute phytotoxicity, suppressing chlorophyll synthesis (263.84 ± 10 mg·L⁻¹ in controls vs. 189.5 mg·L⁻¹ in HAs) and ROL (64.46 mmol O_2_·day⁻¹·g⁻¹ DW in controls vs. 32.23 mmol in HAs). However, 1-month LAs treatment triggered adaptive responses, including chlorophyll recovery (LAs > CK) and compensatory root elongation (HAs > LAs > CK in length/surface area), highlighting the hormetic plasticity of rice. Fe amendments mitigated As toxicity through Fe-plaque formation, which acted as a sustained sink to enrich As in roots while enhancing plant biomass by 1.18–2.39 times under chronic stress. Notably, Fe selectively alleviated radial root suppression (volume/diameter increased by 1.72–1.87 times) but not axial growth, underscoring hierarchical stress prioritization. Paradoxically, Fe reduced root porosity (9.84% vs. 12.1% in non-Fe groups) despite elevating ROL, suggesting rice-specific oxygen transport mechanisms mediated by fine lateral roots lacking aerenchyma. Anatomical analyses confirmed Fe’s role in restoring aerenchyma structure (46.32% vs. 16–17% in controls) and reinforcing lignified barriers, balancing oxygen retention with detoxification.

Our results challenge the traditional porosity–ROL paradigm and emphasize Fe’s dual function: immobilizing As via plaque formation while preserving root metabolic activity. The temporal resilience of ROL under Fe–As co-treatment, reflected by a 1.38–2.83-fold recovery, highlights adaptive oxygen management strategies that are critical for wetland phytoremediation. However, hydroponic conditions may underestimate Fe-plaque efficacy relative to soil systems, necessitating field-scale validation. Future research should explore genetic regulators of root plasticity and microbial synergies in Fe–As interactions. Together, these findings provide a mechanistic foundation for leveraging Fe amendments to enhance crop resilience in As-polluted agroecosystems, bridging remediation science and sustainable agriculture.

## Data Availability

The original contributions presented in the study are included in the article/**Supplementary Material**. Further inquiries can be directed to the corresponding authors.
