# Supplementary material for: Iron-mediated remediation of arsenic-induced suppression of root morphogenesis and radial oxygen loss in wetland plant
Source: Front Plant Sci. 2025 Dec 19;16:1736435. doi: 10.3389/fpls.2025.1736435 (PMC12757279; doi:10.3389/fpls.2025.1736435)
Supplement: Supplementary file 1 [file DataSheet1.docx]

**Iron-Mediated Remediation of Arsenic-Induced Suppression of Root Morphogenesis and Radial Oxygen Loss in Wetland Plant**

Sitong Jiang **^a^**, Zhengyu Zhu **^a^**, Yitong Pan **^b, c^**, Rongrong Shi **^b^**, Zhixi Wang **^b^**, Mohammad Mazbah Uddin **^d^**, Song Wang **^a, *^**, Jingchun Liu **^b, *^**, Kang Mei **^a, b, *^**

**a** *Jiangsu Institute of Marine Resources Development, Jiangsu Key Laboratory of Marine Bioresources and Environment, Jiangsu Ocean University, Lianyungang 222005, China*

**b** *State Key Laboratory of Marine Environmental Science, College of Ocean and Earth Sciences, College of the Environment and Ecology, Xiamen University, Xiamen 361102, China*

**c** *Department of Civil and Environmental Engineering, Princeton University, Princeton, NJ 08544, USA*

**d** *Key Laboratory of the Ministry of Education for Earth Surface Processes & College of Urban and Environmental Sciences, Peking University, Beijing 100089, China*

*Correspondence:*[*kangmei@jou.edu.cn*](mailto:kangmei@jou.edu.cn)*; liujingchun@xmu.edu.cn; wangsong75@163.com*

**Supplementary Information**


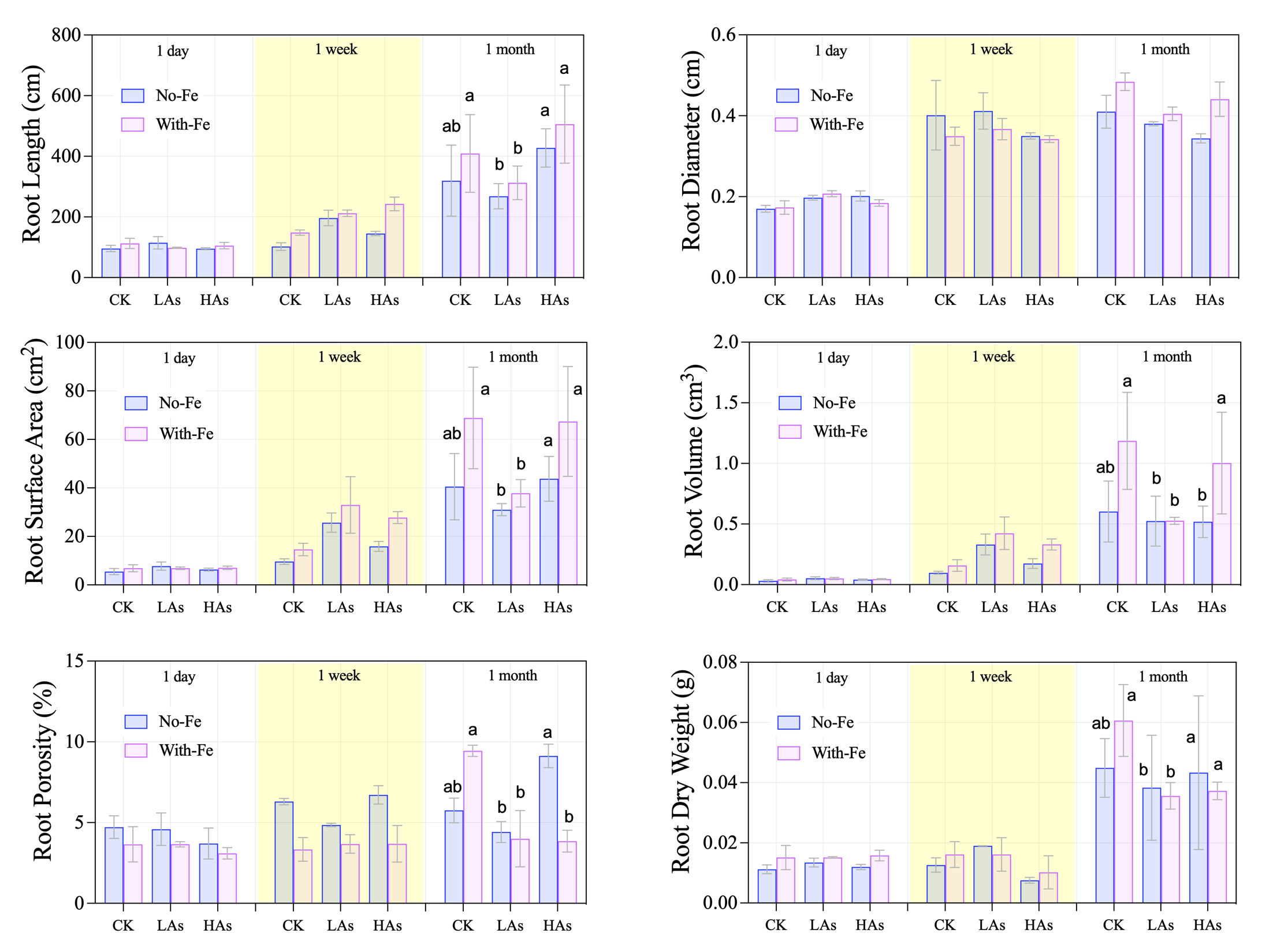


**Fig. S1** Morphological responses of rice *O. Sativa* seedlings under As and Fe treatments


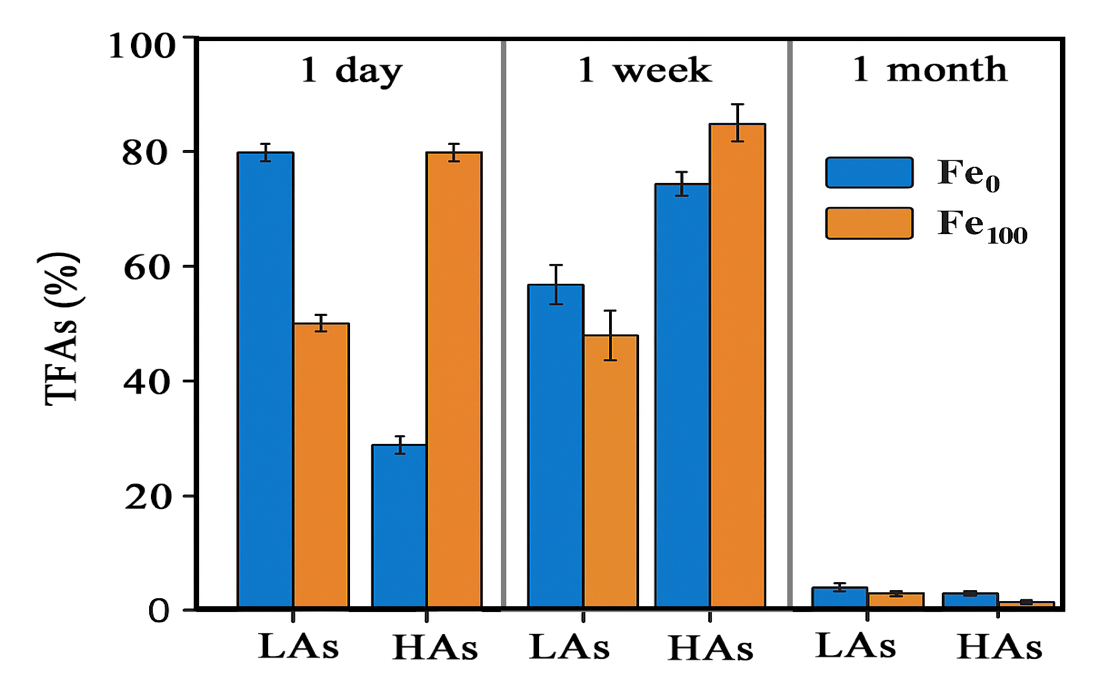


**Fig. S2** Translocation Factor of As translocation in *O. sativa* under As and Fe treatments

**Table S1** Analysis of multi-way ANOVA of *O. sativa* among the concentration of Fe and As in Fe plaque, root, stem and leaf (mean value, n=54)

|  | DCB-As  (mg·kg^-1^) | Root-As  (mg·kg^-1^) | Stem-As  (mg·kg^-1^) | Leaf-As  (mg·kg^-1^) | DCB-Fe  (g·kg^-1^) | Root-Fe  (g·kg^-1^) | Stem-Fe  (g·kg^-1^) | Leaf-Fe  (g·kg^-1^) |
| --- | --- | --- | --- | --- | --- | --- | --- | --- |
| CK | 0.09 c | 0.69 c | 0.04 c | 0.04 c | 2.70 b | 4.94 a | 0.19 b | 0.27 a |
| LAs | 0.22 b | 16.08 b | 0.73 b | 0.50 b | 2.71 b | 5.77 a | 0.16 c | 0.20 b |
| HAs | 0.48 a | 30.32 a | 0.90 a | 0.74 a | 3.87 a | 7.55 a | 0.23 a | 0.15 c |
| Fe Added | ***p*=0.005** | **NS** | **NS** | **NS** | ***p*<0.001** | ***p*=0.011** | **NS** | ***p*=0.057** |
| 1-Day | 0.12 b | 2.50 c | 0.45 c | 0.40 a | 2.84 b | 0.77 b | 0.17 b | 0.23 a |
| 1-Week | 0.40 a | 5.60 b | 0.60 b | 0.39 a | 4.90 a | 9.03 a | 0.26 a | 0.20 b |
| 1-Month | 0.27 a | 38.94 a | 0.62 a | 0.48 a | 1.55 c | 8.47 a | 0.15 c | 0.18 b |
| As*Fe*time | ***p*<0.001** | ***p*<0.001** | ***p*<0.001** | ***p*<0.001** | ***p*<0.001** | ***p*<0.001** | ***p*<0.001** | ***p*<0.001** |

Note: Different letters above indicate significant difference among different treatments at p<0.05 as determined by LSD test.

**Table S2** Hoagland solution composition (100%)

| **Macronutrients** | Stock (mg L^-1^) | **Micronutrients** | Stock (mg L^-1^) |
| --- | --- | --- | --- |
| Ferric tartrate | 5.00 | Boric acid | 2.86 |
| Potassium nitrate | 505.5 | Manganese chloride.4H_2_O | 1.81 |
| Calcium nitrate | 820.75 | Zinc sulphate.7H_2_O | 0.22 |
| Magnesium sulphate | 240.94 | Copper sulphate.5H_2_O | 0.08 |
| Potassium dihydrogen phosphate | 136.1 | Molybdenum trioxide.2H_2_O | 0.02 |
